# Trends in Private Equity Acquisition of Pain Management Practices

**DOI:** 10.1001/jamanetworkopen.2024.51688

**Published:** 2024-12-19

**Authors:** Geronimo Bejarano, James E. Eubanks, Robert T. Braun

**Affiliations:** 1 Department of Health Services, Policy, and Practice, Brown University, Providence, Rhode Island; 2 Department of Orthopaedics and Physical Medicine and Rehabilitation, Division of Physical Medicine and Rehabilitation, Medical University of South Carolina (MUSC), Charleston; 3 Department of Physical Medicine and Rehabilitation, University of Pittsburgh Medical Center (UPMC), Pittsburgh, Pennsylvania; 4 Department of Population Health Sciences, Weill Cornell Medicine, New York, New York

## Abstract

This cross-sectional study of US health care market databases examines geographic patterns in the acquisition of pain management practices by private equity.

## Introduction

Pain has the highest health care spending in the US and is expected to increase with the aging population, which may entice private equity acquisitions of pain management practices.^1^ Private equity has increasingly acquired physician practices and acquisitions are associated with higher spending, utilization of more expensive treatments, and increasing patient volume.^2,3,4^ To our knowledge, there has been no evidence on private equity acquisitions in pain management. This study assesses the trends and geographic variation of private equity acquisitions of pain management practices and physicians.

## Methods

This cross-sectional study was reported following the Strengthening the Reporting of Observational Studies in Epidemiology (STROBE) reporting guideline and approved by the institutional review board at Weill Cornell Medical School with a waiver of informed consent due to deidentified data. Similar to previous studies, private equity acquisitions of pain management practices from 2013 to 2023 were identified using Irving Levin health care market databases and further supplemented with manual searches of press releases and clinic websites in attempts to identify all relevant acquisitions.^2,4^ We found the National Provider Identifier (NPI) of the physician owning the practice, then used the Medicare Data on Provider Practice and Specialty (MD-PPAS) dataset to find the practice’s Tax Identification Number (TIN) and the NPI of all other physicians who billed that TIN.^2^ Practices were defined as pain management practices only when more than half of the physicians in the practice had a primary specialty code in MD-PPAS indicating pain management or interventional pain management. Advanced practice providers (eg, physician assistants) cannot have a pain management specialty in MD-PPAS; therefore, our analysis only included physicians.

We calculated the percentage of pain management practices and physicians that were acquired by private equity stratified by year and state. Using MD-PPAS, we created the denominator as the total number of pain management practices and physicians in those practices each year and state. Analyses were conducted between May 2024 and September 2024 using Stata version 18.0 (StataCorp LLC).

## Results

**Figure 1.**
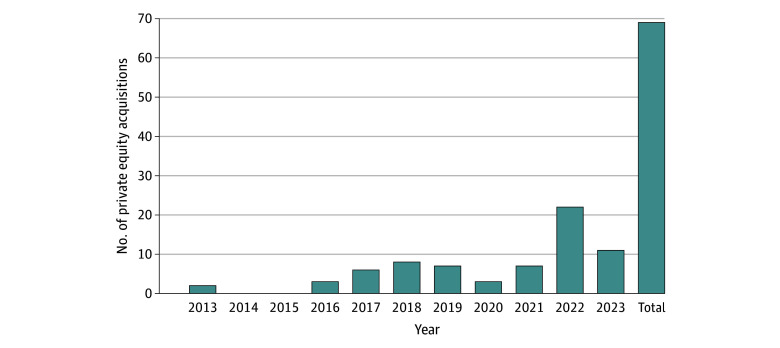
Number of Private Equity Acquisitions of Pain Management Practices by Year Acquisitions were identified using data from Irving Levin health care database. The denominator is the total number of pain management practices in each year.

**Figure 2.**
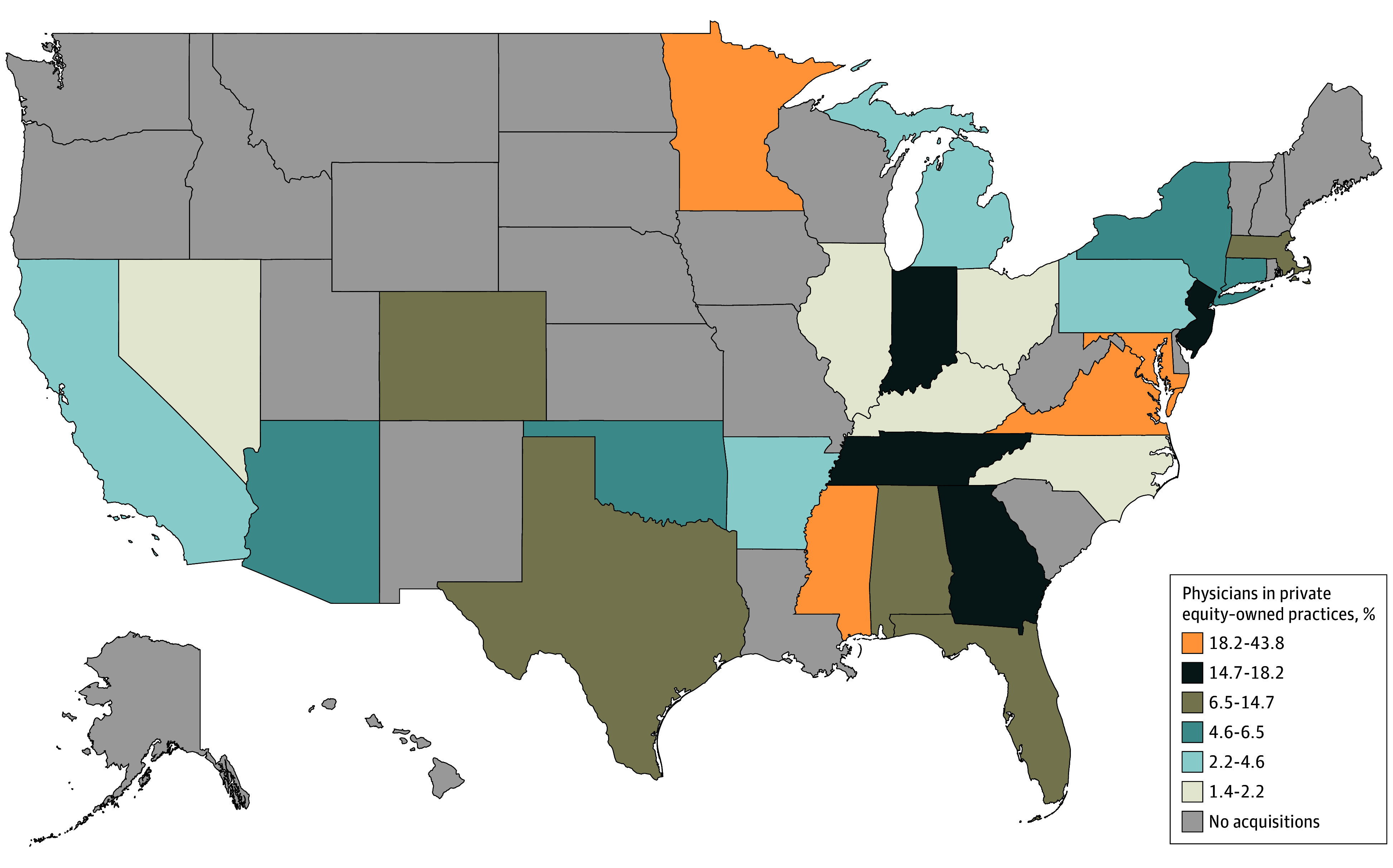
Proportion of Pain Management Physicians Practicing at Private Equity–Acquired Pain Management Practices by State Acquisitions were identified using data from Irving Levin health care database. Data on location of pain management practice were identified using Medicare Data on Provider Practice and Specialty.

There were 69 pain management practices acquired by private equity, which represented 4.3% of the 1588 pain management practices in 2023 (Figure 1). Private equity acquisitions of pain management practices spanned 26 different states, with the most being in Texas (10 [14.5%]), Florida (8 [11.6%]), and New Jersey (6 [8.7%]). As of 2023, 198 of 2241 pain management physicians (8.2%) worked for a private equity–owned pain management practice in the US compared with 0.4% in 2013. The states with the highest percentage of pain management physicians working for private equity–owned pain management practices in 2023 were Virginia (21 of 48 [44%]), Maryland (15 of 59 [25%]), Mississippi (5 of 23 [22%]), and Minnesota (3 of 12 [20%]) (Figure 2).

## Discussion

In this cross-sectional study of private equity acquisitions of pain management practices, we found a rise in acquisitions over the last decade with almost 1 in 10 pain management physicians affiliated with a private equity–owned pain management practice. Private equity penetration in pain management varied widely by geographic region ranging from 24 states with no private equity acquisitions to over 40% of pain management physicians in Virginia, which aligned with the private equity penetration of 6 other specialties found in prior studies.^4^ This high amount of consolidation within certain states poses concerns for private equity to have enough market power to control care delivery of several procedure-based specialties, including pain management. Policymakers and the Federal Trade Commission have taken notice of the harms of increases in both health care consolidation and private equity acquisitions, and there are ongoing efforts to curb their detrimental effects.^5^

Our study is limited in that it may not have captured all private equity acquisitions of pain management practices. Pain management can consist of appropriate care and high-cost treatments that are not recommended by guidelines (eg, epidural steroid injections, spinal cord stimulators, and early imaging).^6^ Monitoring is needed as to ensure any change in pain treatment utilization benefits patients without unnecessary increase of low value care.^2,3^

## Notes

Supplement.
Data Sharing Statement
